# Efficacy of Bloodletting Therapy in Patients with Chronic Idiopathic Urticaria: A Randomized Control Trial

**DOI:** 10.1155/2020/6598708

**Published:** 2020-10-30

**Authors:** Biru Ma, Xiuhua Chen, Yudan Liang, Weiquan Ouyang, Boyan Tang, Fanqi Meng, Xiaohong Quan, Cong Wang, Ying Li, Dacan Chen

**Affiliations:** ^1^Traditional Therapy Clinic, The Second Clinical College of Guangzhou University of Chinese Medicine, Guangzhou 510120, China; ^2^Dermatology Department, The Second Clinical College of Guangzhou University of Chinese Medicine, Guangzhou 510120, China; ^3^Department of Rehabilitation Medicine, Jiangmen Wuyi Hospital of Traditional Chinese Medicine, Jiangmen 529099, China; ^4^Cardiovascular Department, The Second Clinical College of Guangzhou University of Chinese Medicine, Guangzhou 510120, China; ^5^Department of TCM Dermatology, Dermatology Hospital of Southern Medical University, Guangzhou 510095, China; ^6^Department of Rehabilitation Medicine, Peking University Shenzhen Hospital, Shenzhen 518035, China

## Abstract

**Objective:**

To assess the efficacy of bloodletting therapy (acupoint pricking and cupping) in patients with chronic idiopathic urticaria (CIU) in a randomized, control, parallel-group trial.

**Methods:**

A total of 174 patients with CIU enrolled from March 2018 to October 2019 were randomized into three groups: group A treated with bloodletting therapy and ebastine, group B treated with placebo treatment (acupoint pseudopricking and cupping) and ebastine, and group C treated with ebastine only. The intention-to-treat analysis was conducted, and the primary outcome was the effective rate of UAS7 score being reduced to 7 or below after treatment phase.

**Results:**

The effective rates at the end of treatment phase were different among the three groups (*P* < 0.05), which were 73.7% in group A, 45.6% in group B, and 42.9% in group C. Multiple analysis indicated differences between groups A and B (*P* < 0.0125) and groups A and C (*P* < 0.0125) and no difference between groups B and C (*P* > 0.0125). No severe bloodletting therapy-related adverse events were observed.

**Conclusions:**

In this study on patients with CIU, one month of bloodletting therapy combined with ebastine is clinically beneficial compared with placebo treatment combined with ebastine and treatment with ebastine only. Thus, bloodletting therapy can be an effective complementary treatment in CIU. This trial is registered with ChiCTR1800015294.

## 1. Introduction

Chronic idiopathic urticaria, CIU (also known as chronic spontaneous urticaria in recent years), is defined by the appearance of wheals, angioedema, or both for ≥6 weeks due to unknown causes [1]. Chronic urticaria affects about 0.5–1% of the population, and nearly 25% suffers from urticaria at least once during the lifetime [2]. CIU significantly affects the objective functioning and subjective well-being [3–5], resulting in substantial economic burden to patients and society because of its high direct and indirect healthcare costs [3, 6, 7]. The pathogenesis appears to be persistent activation of mast cells in the skin; however, the precise mechanism of mast cell triggering is yet unclear.

The main-stream treatment of CIU targets complete symptom control and modern second-generation H1-antihistamines constitute the first line treatment [1]. However, a meta-analysis reported that the rate of response to standard doses of antihistamine in CIU patients is 38.6% (95% CI 34.7–42.7) [8], similar to that reported more than a decade ago [9]. Moreover, sedation and impaired psychomotor function is reduced with second-generation antihistamines, but it can still occur. [10].

The possibility of a causal influence of emotional distress, especially of stressful life events, on the course of skin diseases has long been postulated [11]. Urticaria and angioedema can lead to significant stress and the converse is also recognized. Acute psychological stress may induce CRH-dependent (corticotropin-releasing hormone) mast cell degranulation [12], and stress-related mechanisms may provide links to CIU [13–15]. Although psychological stress in isolation is unlikely to be the sole trigger, a high frequency of patients with CIU report a stressful event preceding the onset of CIU [16], and patients with CIU experience high rates of anxiety, depression, and somatoform disorders such as fibromyalgia, with half of subjects with CIU being affected by at least one of these conditions [17, 18]. Psychiatric comorbidity appears to significantly increase life quality impairment [19]. Bloodletting therapy is defined as the practice of treating diseases through the removal of a small amount of blood from patients at specific acupoints, collaterals with blood stasis or diseased regions. It has been used since primitive society in China and developed throughout the history of Chinese medicine. Bloodletting therapy has been proved effective in the treatment of generalized anxiety disorder [20], which leads us to speculate the positive influence of this treatment on the stress status of CIU patients and symptom improvement.

Traditional Chinese medicine has a long history in the recognition, identification, and administration of urticaria, which can be traced back to Huangdi Neijing (The Yellow Emperor's Internal Canon of Medicine) [21]. The clinical feature of urticaria with wheals and pruritus coming and going quickly is the manifestation of wind-evil that lurks in and circulates with blood. Hence, in the treatment of urticaria, dispersing wind is the one of the principle methods, and treating blood before wind is an important procedure because when blood flows fluently , wind-evil will resolve spontaneously. Bloodletting therapy is a direct and effective way of regulating blood. It has the potential of being an effective control in chronic urticaria, but the quality of present evidence is low [22–26].

Thus, we designed this randomized controlled trial with appropriate outcome measurements and long-term follow-up, aiming to provide convincing proof for this therapy in the treatment of CIU.

## 2. Materials and Methods

### 2.1. Participants

We recruited CIU patients from the dermatological clinic and the traditional therapy clinic of Guangdong Provincial Hospital of Chinese Medicine (Dade Road General Hospital) via posters and communication media, from March 2018 to October 2019. The diagnostic criteria were in reference to that of chronic spontaneous urticaria, according to the EAACI/GA2 LEN/EDF/WAO Guidelines (2013) [1]. The inclusion criteria were as follows: age: 18–70 years; urticaria activity score (UAS7) ≥ 28; subsequent regular visits. We excluded patients having the disease for ≥2 years; patients who had taken antihistamines within three days and corticosteroids, nonsteroidal anti-inflammatory drugs, or immunosuppressive drugs within one month before enrollment. The autologous serum skin test (ASST) [1] was performed to discern autoimmune urticaria. The eligibility criteria are listed in Table 1.

### 2.2. Sample Size Calculation

A preliminary test showed the effective rate of bloodletting therapy combined with ebastine in treating CIU was 76.8%, and it has been reported that the effective rate of ebastine is 42.9% [27]. Herein, we needed 48 participants in each group to detect the difference between two samples (Stata/MP 13.1, *α* = 0.5, 1−*β* = 0.90). We added 20% to allow for dropouts and, thus, planned to include 58 participants in each group. The total sample size was determined as 174 for the three groups.

### 2.3. Randomization, Masking, and Procedures

Random cards were made and placed in an envelope by specialized staff according to the allocation sequence created using Stata/MP 13.1. Eligible patients were allocated to different groups in the order of the envelope sequence numbers. Except for the therapists, all the other staff and patients were unaware of the assignment groups during data collection.

### 2.4. Intervention

All three groups were prescribed ebastine tablets (Kastine, Industrias Farmaceuticas Almirall, S.A., Import Drug License No. H20140855) 10 mg/d, 28 days. Patients in group A were treated additionally with bloodletting therapy (acupoint pricking and cupping), twice a week for four weeks. Patients in group B were given extra placebo treatment (acupoint pseudopricking and cupping), twice a week for four weeks.

### 2.5. Bloodletting Therapy (Acupoint Pricking and Cupping)

Acupoints: Danshu (BL19, bilateral), Geshu (BL17, bilateral). After locating the acupoints, the therapist sterilized the skin and pricked three times on each acupoint with a disposable blood collection needle (SteriLance, Suzhou SteriLance Medical Device Co., Ltd.). All three pricks should be performed precisely within 1 mm to the exact acupoint location. Then, cupping was performed on the pricked acupoints and removed after 5 min. The skin was cleaned and sterilized at the end of the treatment. The volume of the blood let out should be controlled around 1 mL per acupoint.

### 2.6. Placebo Treatment (Acupoint Pseudopricking and Cupping)

Acupoints: Danshu (BL19, bilateral), Geshu (BL17, bilateral). The procedure was exactly the same as that of group A, but the handle of an ordinary acupuncture needle was used instead, and the handle head was gently pricked to make the patient feel the stimulation without piercing the skin.

### 2.7. Outcomes and Data Collection

In this trial, the primary outcome was the effective rate of UAS7 being reduced to 7 or below after treatment phase. The UAS is based on the assessment of key urticaria symptoms (wheals and pruritus). The UAS score ranges from 0–6. High score indicates severe activity of urticaria. As urticaria symptoms frequently change in intensity, the overall disease activity is best measured by advising patients to document 24 h self-evaluation scores for several days. UAS7 is the accumulation of UAS scores (0–42) self-collected in seven days. Secondary outcomes included the effect rate of UAS7 being reduced to 14 or below during follow-ups, Symptom Check List 90 (SCL90), Dermatology Life Quality Index (DLQI), and serum IgE level. Patients were instructed to document everyday UAS score one week before treatment till the end of follow-up at week eight. SCL90 and DLQI scores were collected before treatment, once a week during the treatment phase and at the end of weeks five and eight during follow-up. Serum IgE level tests were conducted before and at the end of treatment phase. The trial measurements were unchanged after trial commencement. The trial physicians or therapists performed active surveillance of side effects and adverse events from baseline to five weeks using the Adverse Events Surveillance Form, which were reported in this study. We also monitored compliance using a Patient Compliance Form. Adverse events and compliance were monitored and cross-checked against the patient's clinical notes.

### 2.8. Statistical Analysis

We compared the effective rates after treatment phase (week four, UAS7 ≤ 7) and at the end of follow-ups (weeks five and eight, UAS7  ≤ 14) among groups by using *χ*^2^ test (*α* = 0.05) and performed multiple comparisons between groups with adjusted *α*′(*α*′ = *α*/(2(*k* − 1))), *α*′ = 0.0125). In this part, we conducted both the intention-to-treat analysis and the per-protocol analysis and dealt with the missing values of dropout or lost-to-follow-up cases by method of last observation carried forward (LOCF). In addition, in the analyses of scores of UAS7, SCL90, and DLQI, we performed linear mixed effects models in the intention-to-treat analysis and variance analysis for repeated measurements in the per-protocol analysis. Adverse events were presented descriptively. Two statisticians blinded to the treatment group independently carried out the intention-to-treat analyses and per-protocol analyses with a software package SPSS (version 18.0).

## 3. Results and Discussion

### 3.1. Patients

**Figure 1:**
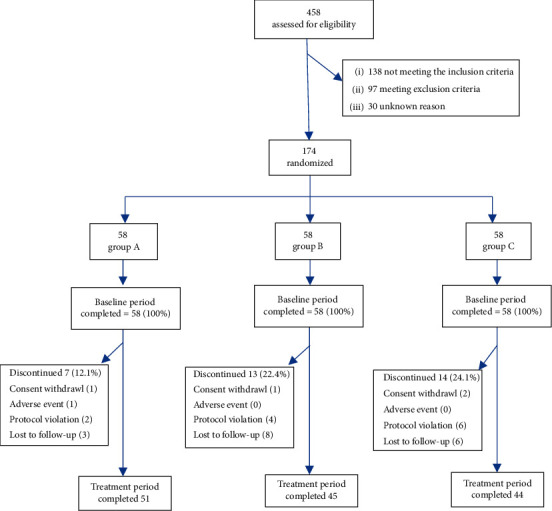
Flowchart showing trial group assignments, loss to follow-up, and treatment completion.

Figure 1 summarizes the trial group assignments, loss to follow-up, treatment completion, and protocol deviations. Of the 458 patients assessed for eligibility, 174 underwent randomization. In total, 170 randomized patients were started on the treatment and were included in the intention-to-treat analysis and safety description. Of these, 140 patients who went through the trial without protocol violation were included in the per-protocol analysis.  Table 2 shows the baseline characteristics of participants. We did not consider any differences between the treatment groups to be relevant.

### 3.2. Outcomes

The effective rates (UAS7 ≤ 7) at the end of treatment phase (week four) were significantly different among the three groups (*χ*^2^ = 13.308, *P*=0.001), which were 73.7% in group A, 45.6% in group B, and 42.9% in group C. Multiple analysis indicated significant differences between groups A and B (*χ*^2^ = 9.330, *P*=0.004) and groups A and C (*χ*^2^ = 11.050, *P*=0.001) and no significant difference between groups B and C (*χ*^2^ = 0.087, *P*=0.850). The effective rates (UAS7 ≤ 14) during the follow-up periods were as follows: at the end of week five, significant difference was observed among the three groups (*χ*^2^ = 11.059, *P*=0.004), that is, 73.7% in group A, 43.9% in group B, and 51.8% in group C; at the end of week eight, the effective rates were 61.4% in group A, 22.8% in group B, and 21.4% in group C, also with significant difference among the three groups (*χ*2 = 25.621, *P* < 0.001). Results of further multiple comparisons were similar to those of week four (Table 3). Results of the per-protocol analysis were in consistency with those of the intention-to-treat analysis (Table 4).

**Figure 2:**
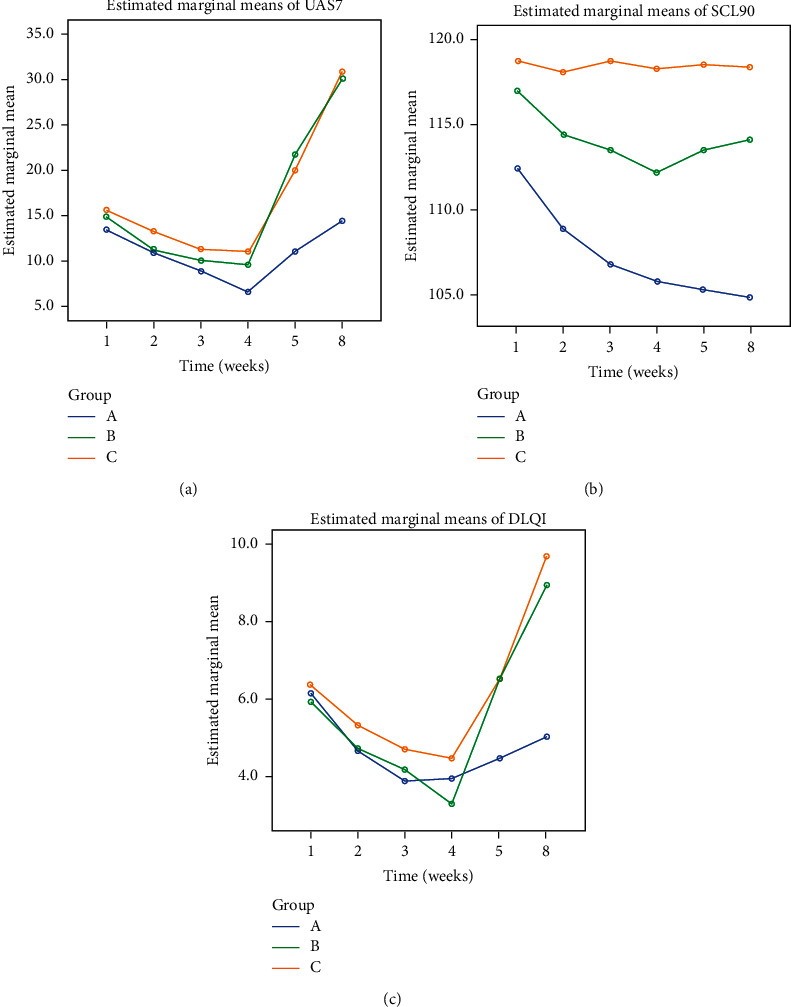
Profile of time and grouping factors.

In the linear mixed effects models analyses, the hypothesis tests of the selected repeated measurement structure met the requirements and the results converged, indicating that the models were reasonable (Table 5). Analysis results indicated that treatment effects were statistically significant in all UAS7, SCL90, and DLQI scores (Table 6). UAS7 pairwise comparisons showed that significant differences between groups A and B occurred at weeks five (−10.76, 95%CI (−14.05, −7.47), *P* < 0.001) and eight (−15.51, 95%CI (−18.15, −13.76), *P* < 0.001), and that significant differences between groups A and C occurred at weeks four (−4.36, 95%CI (−7.68, −1.044), *P*=0.010), five (−8.92, 95%CI (−12.22,−5.62), *P* < 0.001), and eight (−16.41, 95%CI (−19.06, −13.76), *P* < 0.001). No significant difference in UAS7 was observed between groups B and C at all time periods (Table 7). Scores of SCL90 differed significantly between groups A and B from weeks one to eight (*P* < 0.05), except week four (*P*=0.058), and between groups A and C at all time periods (*P* < 0.01, Table 8). Significant differences in DLQI occurred only at weeks five and eight between groups A and B and groups A and C (*P* < 0.001, Table 9). In the per-protocol analysis, data before treatment were taken as covariates, and the results of variance analysis of repeated measurements were consistent with those of linear mixed effects models in the intention-to-treat analysis (Figure 2).

Serum IgE levels did not differ significantly among the three groups before and after treatment (Table 10).

### 3.3. Adverse Events

Six patients (3.53%, *n* = 170) had drowsiness during the treatment period and were advised to take ebastine before sleep. One patient (0.59%, *n* = 170) in group A had dizziness after the initial two times of bloodletting therapy and discontinued the trial voluntarily. No other adverse events were observed.

### 3.4. Principal Findings

In this study on patients with CIU, four weeks of combined treatment with bloodletting therapy (acupoint pricking and cupping) and ebastine was clinically beneficial compared with combined treatment with placebo treatment (acupoint pseudopricking and cupping) and ebastine and single treatment with ebastine, which manifested at both the end of treatment phase and follow-ups. The secondary analyses supported this finding. No severe bloodletting therapy-related adverse event was observed.

### 3.5. Clinical Relevance

CIU is a common and debilitating allergic condition; however, its pathogenesis is yet unknown, and the treatment is tenuous. In this trial, a second-generation H1-antihistamine, ebastine, was prescribed as the first remedy. The effective rate of ebastine was 42.9% at week four and was 51.8% at week five; nonetheless, the effective rate was only 21.8% at week eight, proximate to that of the previous study, reflecting the weakness of second-generation H1-antihistamine in the long-term control of CIU. Previous studies on CIU, conducted over three and twelve months, reported that approximately 65% of the patients treated with 10 mg ebastine experienced major improvement at the end of treatment according to the global patient/physician ratings [28, 29]. Different response standard and medication period may lead to different response rate in this trial. There is general agreement regarding the use of higher doses of second-generation antihistamines as a second-step therapy in patients with severe, recalcitrant CIU for whom the standard dose is not effective. However, this agreement is mainly supported by isolated clinical studies. British guidelines give a grade B for the recommendation to use higher doses of antihistamines [10]. Likewise, Japanese guidelines give a recommendation level B–C1 and an evidence level II, V for increasing the dose of antihistamines up to twice the recommended amount when no response is achieved at standard doses [30]. In contrast, American guidelines agree that the data are limited and conflicting regarding the recommendation of up-dosing antihistamines for patients who are not responsive to standard doses [31]. A meta-analysis finds no differences in wheal number or response rates, and only significant differences with respect to the control of pruritus, with a low improvement magnitude (0.13 on a scale of 0–3) [8]. In brief, we should be cautious about up-dosing antihistamines and further high-quality studies on up-dosing are really needed.

In this study, bloodletting therapy combined with ebastine showed better curative effects from the other two groups for medium to severe CIU (baseline UAS7 ≥ 28) at end of treatment phase and follow-ups, which were 73.7% at weeks four (UAS7 ≤ 7) and five (UAS7 ≤ 14), and a remaining 61.4% at week eight (UAS7 ≤ 14), respectively, with significantly lower UAS7 score at weeks one and four after treatment termination but without severe adverse effect. It may provide positive solution to refractory CIU cases which do not respond to standard doses of second-generation H1-antihistamines. Notably, no differences were detected between groups B and C, indicating that cupping was merely an operation that helped bloodletting rather than an effective manipulation in symptom control, while bleeding was the crucial procedure.

Bloodletting therapy help reduce the disease activity on the aspects of severity and recurrence frequency, both of which are directly related to the improvement of the psychological status (SCL90) and life quality (DQLI). Stress is an independent factor in the development and exacerbation in CIU, and bloodletting therapy on Sihua points (the combination of Geshu and Danshu points) has been proved to be effective in the treatment of general anxiety disorder [20]. We observed significant difference of SCL90 prior to the occurrence of significant difference of UAS7 between groups. Presumably, the improvement in SCL90 by bloodletting on Sihua points is beneficial to the alleviation of CIU symptom, forming a virtuous circle. Another beneficial factor needed consideration is patient education. Despite not being included in the intervention plan, the concepts of the complexity of CIU pathology and myriad causes and the importance of avoidance were relayed to the patients through natural physician–patient communication. This might be the missing link in the CIU management algorithm in guidelines.

CIU is an inflammatory disease [32, 33], where histamine plays a key role, but one of the players in a much complex disease. A previous study observed elevated interferon-γ (IFN-*γ*) level and lowered interleukin 4 (IL-4) and IgE levels in CU patients treated with Back-Shu point acupuncture combined with pricking and cupping therapy and presumed that the therapy might correct the Th1/Th2 imbalance and reduce mast cell activation, thereby improving the CIU symptoms [34]. In addition, bloodletting therapy is shown to alleviate CIU by reducing serous leukotriene B4 (LTB4) and prostaglandin D2 (PGD2) levels [25]. Ear bloodletting therapy can reduce the expression level of interleukin-17 (IL-17) and interleukin-23 (IL-23) [35] and the expression level of prostaglandin E2 (PGE2), thromboxane B2 (TXB2), leukotriene B4 (LTB4), and 6-keto-prostaglandin F1-*α* (6-k-PGF1-*α*) [36] in patients with eczema. However, we did not detect similar changes in the IgE level among groups. Further research is needed on the effective mechanism of bloodletting therapy in treating CIU. Immunomodulatory mechanisms as well as stress-related mechanisms may provide new perspective.

### 3.6. Limitations

Nevertheless, the present study has some limitations. The main limitation was that we could not rule out the possibility that some patients administered additional drugs or avoided ebastine without informing us, which might influence the difference in outcomes between the treatment groups. In order to reduce this problem, we encouraged the patients to register the drugs in patients' notes and give us a genuine record of medication and symptom evaluation. Secondly, this was a single-centre trial, and participants had a propensity for complementary methods, and we enrolled patients who had endured only two years of the disease, which may cause sampling bias.

## 4. Conclusions

Bloodletting therapy is an effective and safe complementary treatment in CIU. Further studies should focus on the underlying mechanism and large-scale multicentre standard control for its general applicability.

## Figures and Tables

**Table 1:** | Eligibility criteria.

Eligibility criteria	Details
Diagnostic criteria	Spontaneous wheals and/or angioedema >6 weeks;
	Average wheal duration <24 h;
	Exclusion of physical urticaria and other chronic urticaria types.

Inclusion criteria	Age 18–70 years;
	Wheals and pruritus present almost daily, UAS7 ≥ 28;
	Regular subsequent visits.

Exclusion criteria	Usage of antihistamines within three days or corticosteroids, nonsteroidal anti-inflammatory drugs, and immunosuppressive drugs within one month before inclusion;
	Course of disease >2 years;
	Autoimmune urticaria;
	Autoimmune diseases;
	Allergic to ebastine;
	Pregnant or breast-feeding women;
	Thrombocytopenia and coagulation disturbance;
	Abnormal liver and kidney function;
	Trauma/infection;
	Accompanied by fever, dizziness, headache or vomiting;
	Cardiovascular and cerebrovascular disease, diabetes, kidney disease, abnormal bone metabolism, and acute attack of asthma;
	Mental disorder;
	Ulcer, infection, or scar on treated skin;
	Mistakenly enrolled;
	Severe systemic symptoms or other serious diseases found during treatment;
	Intermittent treatment;
	Dropout or withdrawal voluntarily.

**Table 2:** Baseline characteristics of participants.

	A	B	C	Kruskal–Wallis H test	Fisher's exact test
	*n* = 58	*n* = 58	*n* = 58		
Age (mean(SD))	39.83 (1.88)	40.57 (1.91)	40.62 (1.91)	*P*=0.979	
Course (mean(SD))	16.78 (1.18)	13.88 (1.26)	13.91 (1.25)	*P*=0.111	
Sex (male/female (%))	19/39	11/47	11/47		*P*=0.163
	(32.8/67.2)	(19.0/81.0)	(19.0/81)		

**Table 3:** Primary outcomes of treatment groups and treatment comparisons (ITTA^*∗*^).

Outcomes	Group A	Group B	Group C	*χ*2 test ^*∗∗*^	Multiple comparison^*∗∗∗*^		
	*n* = 57 (%)	*n* = 47 (%)	*n* = 56 (%)	(*P* value)	A&B	A&C	B&C
					*χ*2 (*P* value)	*χ*2 (*P* value)	*χ*2 (*P* value)
Week 4	42	26	24	13.308	9.330	11.050	0.087
UAS7 ≤ 7	(73.7)	(45.6)	(42.9)	(0.001)	(0.004)	(0.001)	(0.850)
Week 5	42	25	29	11.059	10.462	5.800	0.711
UAS7 ≤ 14	(73.7)	(43.9)	(51.8)	(0.004)	(0.002)	(0.020)	(0.454)
Week 8	35	13	12	25.621	17.417	18.581	0.031
UAS7 ≤ 14	(61.4)	(22.8)	(21.4)	(<0.001)	(<0.001)	(<0.001)	(1.000)

^*∗*^Intention-to-treat analysis; ^*∗∗*^bilateral, *α* = 0.05; ^*∗∗∗*^bilateral *α*′=0.0125.

**Table 4:** Primary outcomes of treatment groups and treatment comparisons (PPA^*∗*^).

Outcomes	Group A, *n* = 51 (%)	Group B, *n* = 45 (%)	Group C, *n* = 44 (%)	*χ*2 test ^*∗∗*^	Multiple comparison^*∗∗∗*^		
				(*P* value)	A&B	A&C	B&C
					*χ*2 (*P* value)	*χ*2 (*P* value)	*χ*2 (*P* value)
Week 4	38	21	19	11.596	7.824	9.659	0.109
UAS7 ≤ 7	(74.5)	(46.7)	(44.2)	(0.003)	(0.007)	(0.003)	(0.832)
Week 5	37	15	18	16.827	14.809	9.701	0.547
UAS7 ≤ 14	(72.5)	(33.3)	(40.9)	(<0.001)	(<0.001)	(0.003)	(0.515)
Week 8	30	3	1	52.277	20.829	34.362	1.001
UAS7 ≤ 14	(58.8)	(6.7)	(2.3)	(<0.001)	(<0.001)	(<0.001)	(0.616)

^*∗*^Per-protocol analysis; ^*∗∗*^bilateral, *α* = 0.05; ^*∗∗∗*^bilateral *α*′=0.0125.

**Table 5:** Estimation of covariance parameters in linear mixed effects models.

Dependent variable	Parameter		Estimation	SD	Wald Z	*P*	95% CI	
							Lower limit	Upper limit
UAS7	Repeated	CS diagonal offset	53.88	2.82	19.11	<0.01	48.62	59.69
	Measurement	CS covariance	34.22	4.95	6.91	<0.01	24.51	43.92

SCL90	Repeated	CS diagonal offset	47.57	2.50	19.05	<0.01	42.92	52.73
	Measurement	CS covariance	145.88	17.07	8.55	<0.01	112.41	179.35

DLQI	Repeated	CS diagonal offset	6.97	0.36	19.11	<0.01	6.30	7.72
	Measurement	CS covariance	4.71	0.67	7.01	<0.01	3.39	6.02

**Table 6:** Fixed effects tests of linear mixed effects models.

Dependent variable	Source of covariance	Numerator degrees of freedom	Denominator degrees of freedom	F	*P*
UAS7	Intercept	1	485.497	0.146	0.702
	Group	2	488.671	2.946	0.054
	Time	1	759.500	0.005	0.943
	Group ^ ^*∗*^^ time	2	764.056	40.204	<0.001
	Baseline	1	482.538	6.224	0.013
	Time ^*∗*^ baseline	1	755.909	4.599	0.032

SCL90	Intercept	1	245.446	14.624	<0.001
	Group	2	244.683	2.892	0.057
	Time	1	743.885	0.001	0.976
	Group ^*∗*^ time	2	741.350	8.357	<0.001
	Baseline	1	245.657	532.742	<0.001
	Time ^*∗*^ baseline	1	744.661	1.406	0.236

DLQI	Intercept	1	470.664	1.953	0.163
	Group	2	475.203	2.165	0.116
	Time	1	760.496	4.718	0.030
	Group ^*∗*^ time	2	764.635	20.116	<0.001
	Baseline	1	470.404	179.695	<0.001
	Time ^*∗*^ baseline	1	763.768	0.720	0.397

**Table 7:** Means of UAS7 and treatment comparisons for all time periods.

	Group A		Group B		Group C		Pairwise comparison^∗^		
Time	N	Mean (SD)	N	Mean (SD)	N	Mean (SD)	A-B (95%CI)	A-C (95%CI)	B-C (95%CI)
							*P* value	*P* value	*P* value
Baseline	58	34.29	58	32.48	58	32.72			
		(0.80)		(0.73)		(0.72)			

Week 1	57	14.37	57	14.90	56	14.89	−1.52	−1.404	0.12
		(10.46)		(9.58)		(9.88)	(−5.08, 2.03)	(−4.98,2.17)	(−3.43, 3.67)
							0.400	0.493	0.948

Week 2	57	11.51	52	11.52	49	12.92	−0.70	−1.78	−1.08
		(8.82)		(9.48)		(9.59)	(−4.14, 2.75)	(−5.26, 1.70)	(−4.64, 2.47)
							0.691	0.315	0.549

Week 3	53	9.55	50	10.04	45	11.22	−1.05	−2.05	−1.00
		(8.11)		(8.94)		(8.91)	(−4.41, 2.30)	(−5.48, 1.38)	(−4.47, 2.48)
							0.536	0.239	0.571

Week 4	51	6.765	45	9.43	44	10.91	−2.98	−4.36	−1.38
		(6.03)		(9.04)		(9.05)	(−6.28, 0.338)	(−7.68, −1.044)	(−4.80, 2.02)
							0.078	0.010	0.422

Week 5	51	11.63	45	21.33	44	19.77	−10.76	−8.92	1.84
		(8.98)		(8.91)		(8.20)	(−14.05, −7.47)	(−12.22, −5.62)	(−1.56, 5.23)
							<0.001	<0.001	0.286

Week 8	51	15.29	45	29.49	44	30.61	−15.51	−16.41	−0.90
		(9.53)		(7.04)		(6.36)	(−18.15, −13.76)	(−19.06, −13.76)	(−3.62,1.83)
							<0.001	<0.001	0.516

^*∗*^Based on estimated marginal means.

**Table 8:** Means of SCL90 and treatment comparisons for all time periods.

Time	Group A		Group B		Group C		Pairwise comparison^*∗*^A		
	N	Mean (SD)	N	Mean (SD)	N	Mean (SD)	A-B (95%CI)	A-C (95%CI)	B-C (95%CI)
							*P* value	*P* value	*P* value
Baseline	58	128.21	58	120.86	58	121.09			
		(4.07)		(4.00)		(3.93)			

Week 1	57	116.90	57	115.83	56	117.98	−4.50	−6.10	−1.61
		(25.38)		(29.82)		(28.17)	(−8.56, −0.43)	(−10.18, −2.03)	(−5.67, −2.45)
							0.030	0.004	0.435

Week 2	57	113.60	52	114.25	49	115.16	−4.85	−7.28	−2.43
		(24.19)		(30.39)		(26.31)	(−9.55, −0.14)	(−12.07, −2.48)	(−7.31, 2.44)
							0.044	0.003	0.326

Week 3	53	110.68	50	110.64	45	115.89	−5.56	−10.69	−5.14
		(22.92)		(26.54)		(28.41)	(−10.78, −0.33)	(−16.06, −5.33)	(−10.55, 0.274)
							0.037	<0.001	0.063

Week 4	51	109.61	45	1110.07	44	116.02	−5.37	−11.96	−6.60
		(28.00)		(26.37)		(28.41)	(−10.93, 0.194)	(−17.56, −6.37)	(−12.33, −0.86)
							0.058	<0.001	0.025

Week 5	51	109.20	45	111.33	44	116.25	−7.31	−12.58	−5.26
		(22.51)		(27.93)		(27.34)	(−12.71, −1.922)	(−18.00, −7.15)	(−10.83, 0.30)
							0.008	<0.001	0.063

Week 8	51	109.00	45	111.82	44	115.91	−8.29	−12.61	−4.31
		(222.51)		(32.36)		(32.64)	(−15.52, −1.47)	(−19.48, −5.74)	(−11.36, 2.73)
							0.018	<0.001	0.228

^*∗*^Based on estimated marginal means.

**Table 9:** Means of DLQI and treatment comparisons for all time periods.

Time	Group A		Group B		Group C		Pairwise comparison^*∗*^		
	N	Mean (SD)	N	Mean (SD)	N	Mean (SD)	A-B (95%CI)	A-C (95%CI)	B-C (95%CI)
							*P* value	*P* value	*P* value
Baseline	58	11.46	58	9.64	58	9.64			
		(0.84)		(0.86)		(0.84)			

Week 1	57	7.18	57	5.79	56	5.93	0.12	−0.02	−0.14
		(5.64)		(5.33)		(4.58)	(−1.01, 1.26)	(−1.17, 1.12)	(−1.28, 1.00)
							0.832	0.970	0.802

Week 2	57	5.54	52	4.89	49	5.14	−0.19	−0.46	−0.27
		(4.67)		(5.35)		(4.72)	(−1.51, 1.12)	(−1.80, 0.88)	(−1.62, 1.09)
							0.772	0.497	0.698

Week 3	53	4.76	50	4.10	45	4.64	−0.30	−0.69	−0.39
		(4.44)		(4.69)		(4.50)	(−1.58, 0.99)	(−2.01, 0.63)	(−1.72, 0.94)
							0.650	0.306	0.562

Week 4	51	4.9	45	2.87	44	4.16	−0.77	−0.48	−1.25
		(3.94)		(3.83)		(4.36)	(−0.53, 2.06)	(−17.80, 0.82)	(−2.57, 0.08)
							0.245	0.466	0.066

Week 5	51	5.28 (4.13)	45	5.98	44	6.11	−1.82	−1.81	0.00
				(4.73)		(4.78)	(−3.08, −0.55)	(−3.08, −0.55)	(−1.29, 1.30)
							0.005	0.005	0.998
	51	6.14	45	8.18	44	9.16	−3.54	−4.40	−0.86
		(4.16)		(5.53)		(6.20)	(−4.54, −2.54)	(−5.40, −3.40)	(−1.88, 0.16)
							<0.001	<0.001	0.099

^*∗*^Based on estimated marginal means.

**Table 10:** Serum IgE levels before and after treatment.

IgE	Group A	Group B	Group C	K–Wallis H test
Baseline	222.50	354.78	350.35	*P*=0.515
	(60.97)	(219.98)	(207.54)	

Week 4	228.17	342.84	330.87	*P*=0.552
	(64.61)	(183.26)	(189.00)	

## Data Availability

The data used in this paper are available upon reasonable request from the corresponding author.
